# Capacity and use of diagnostics and treatment for patients with severe acute respiratory infections in the pre-COVID-19 era in district and provincial hospitals in Viet Nam

**DOI:** 10.5365/wpsar.2021.12.4.835

**Published:** 2021-11-30

**Authors:** Vu Quoc Dat, Nguyen The Hung,, Kim Bao Giang, Hieu Quang Vu, Satoko Otsu

**Affiliations:** a Department of Infectious Diseases, Hanoi Medical University, Hanoi, Viet Nam.; b National Hospital of Tropical Diseases, Hanoi, Viet Nam.; c Institute for Preventive Medicine and Public Health, Hanoi Medical University, Hanoi, Viet Nam.; d World Health Organization Viet Nam Country Office, Hanoi, Viet Nam.; # These authors contributed equally to this work.

## Abstract

**Objective:**

To describe the burden of severe acute respiratory infection (SARI) and the infrastructure and current practices of SARI management in hospitals in Viet Nam.

**Methods:**

We conducted a short observational study at critical care units (CCUs) in 32 district hospitals and 16 provincial hospitals in five provinces in Viet Nam from March to July 2019. We collected data on hospital equipment and medicines used in SARI management. At the patient level, data were collected for 14 consecutive days on all patients presenting to CCUs, including information on demographics, intervention and treatment within 24 hours of CCU admission and 7-day outcome.

**Results:**

There were significant differences between district and provincial hospitals in the availability of microbial culture, rapid influenza diagnostic tests, inflammatory markers and mechanical ventilation. Among 1722 eligible patients admitted to CCUs, there were 395 (22.9%) patients with SARI. The median age of SARI patients was 74 (interquartile range: 58–84) years; 49.1% were male. Although systemic antibiotics were available in all hospitals and were empirically given to 93.4% of patients, oseltamivir was available in 25% of hospitals, and only 0.5% of patients received empiric oseltamivir within 24 hours of admission. The 7-day mortality was 6.6% (26/395). Independent factors associated with 7-day mortality were septic shock and requiring respiratory support within 24 hours of admission.

**Discussion:**

SARI is a major burden on CCUs in Viet Nam. Barriers to delivering quality care include the limited availability of diagnostics and medication and non-protocolized management of SARI in CCUs.

Severe acute respiratory infection (SARI) remains a substantial burden on health care systems worldwide, with more than 2.5 million deaths in 2017, when it was ranked the fourth leading cause of death for all ages globally. (*1*) During the first two decades of the 21st century, the emergence of novel respiratory infections such as severe acute respiratory syndrome virus (SARS), avian influenza, Middle East respiratory syndrome (MERS) and novel H1N1 pandemic influenza posed significant threats to humans, particularly in Asia. (*2*) In December 2019, the novel severe acute respiratory syndrome coronavirus 2 (SARS-CoV-2), the pathogen that causes coronavirus disease 2019 (COVID-19), was first identified in Wuhan, China; it rapidly spread across the world and was declared a pandemic in March 2020. (*3*)

Lower- and middle-income countries (LMICs) are more vulnerable to infectious diseases, especially epidemic- and pandemic-prone SARI, owing to the lack of preparedness required for critical care services, including health care worker training, infrastructure and supplies. (*4*, *5*) Delivering high-quality care in critical care units (CCUs) in LMICs is challenged by a relative lack of epidemiologic data, context-specific effective interventions and resources. (*6*-*8*) In addition, during outbreaks, health care systems and CCUs are under a greater burden. (*9*, *10*) However, protocolization of critical care in LMICs is limited, and the use of available diagnostics and treatment in this setting is not well known. (*11*)

Viet Nam is an LMIC that has experienced many outbreaks of emerging infectious respiratory diseases such as SARS-CoV, avian influenza A(H5N1) and SARS-CoV-2. (*12*-*14*) Most of the SARI studies in Viet Nam mainly describe clinical and pathological characteristics but give little information about the concordance between clinical management capacity and the availability of medical supplies in association with patient outcome. (*15*-*17*) Our previous assessment of health care infrastructure capacity to respond to SARI indicated enormous limitations on relevant structural and human resources in selected district and provincial hospitals in Viet Nam. (*18*) This study describes current practices in SARI case management and the burden to CCUs on medical resources in district and provincial hospitals in Viet Nam in the months leading up to the COVID-19 pandemic.

## Methods

### Study design

This was a multicentre, prospective, observational study to evaluate the management and outcomes of patients with SARI who were admitted to CCUs in Viet Nam. As of 2019, Viet Nam had 63 provinces divided into six administrative regions, with a population of 96.5 million. (*19*) Per 10 000 inhabitants, Viet Nam had 28.5 hospital beds and 8.8 medical doctors. (*19*) In this study, we used convenience sampling to select five provinces from different administrative regions. In each province, we invited all hospitals at the provincial and district levels to participate in the study. In each participating hospital, we excluded surgical CCUs and paediatric CCUs. Between March and July 2019, all participating hospitals underwent a 14-day observational period. During the first 7 days, all patients aged (*3*)18 years admitted to the eligible CCUs were enrolled in the study, and all were observed for outcomes for 7 days from their enrolment.

### Data collection

SARI cases were defined as: 1) a history of fever or measured fever (*3*)38 °C, 2) cough, 3) symptom onset within the past 10 days and 4) requiring hospitalization. (*20*) Patient outcomes were evaluated at 7 days after admission to the CCU, or when the patient was discharged or transferred to another hospital, whichever came first.

We collected data related to clinical management of SARI in the CCUs from hospital administration records and the patients’ medical records. Data from hospital administration records included information on the availability or use of laboratory tests and medication given to the patients to manage SARI and sepsis that follow international and national guidelines. (*21*, *22*) Demographic characteristics, onset of symptoms and medical history were collected using a modified standardized questionnaire on arrival to the CCU. (*23*) Relevant comorbidities included chronic cardiac disease, chronic renal disease, chronic liver disease and chronic respiratory disease, according to World Health Organization definitions of pre-existing conditions associated with increased risk of severe influenza or death. (*24*) We calculated the quick sequential organ failure assessment (qSOFA) score within the first 24 hours of admission, giving one point for each of three criteria: respiratory rate (*3*)22 breaths/minute, altered mentation and systolic blood pressure £100 mmHg. (*22*) For each patient, information on relevant treatments and interventions during the first 24 hours of admission and early mortality (within 7 days of CCU admission) was also extracted from patients’ medical records.

### Ethics

This study was approved by the Institutional Review Board of Hanoi Medical University (approval number 59/GCN-DDNCYSH-DHYHN). All participants or legal guardians were informed about the study’s purpose and gave their verbal consent for use of their data. The need for written consent was waived by the Institutional Review Board because the data collected were extracted from medical records as part of routine clinical care, with minimal risk of harm to the participants.

### Statistical analysis

Data collected on paper case report forms were entered into an electronic database (EpiData, Odense, Denmark). The proportion of patients who received laboratory tests was calculated as the number of patients who received a test divided by the total patients admitted to all CCUs in which the test was available. Statistical analysis was performed using R software version 3.6.1. All categorical data were calculated as frequencies and compared using χ^2^ or Fisher’s exact test, as appropriate. Continuous variables were given as medians with interquartile range (IQR), and comparisons between groups were performed using the Mann–Whitney U test or Kruskal–Wallis test, as appropriate. Cox proportional hazards regression was used to identify variables that predicted 7-day mortality. *P*-values < 0.05 were considered statistically significant.

## Results

Of the 51 hospitals invited to participate in the study, 48 responded (94% response rate). A total of 1759 patients were admitted to the 48 participating CCUs between March and July 2019 (**Appendix Fig. 1**). We excluded from this analysis 37 (2.1%) patients with no information on diagnosis or date of symptom onset. Among the 1722 eligible patients admitted to CCUs, 395 (22.9%) met the definition of SARI and 1327 (77.1%) had other diagnoses (non-SARI) on admission. The numbers of patients presenting to district hospital CCUs and provincial hospital CCUs were 929 (53.9%) and 793 (46.1%), respectively. The proportion of SARI cases among patients admitted to district CCUs was significantly higher than among those admitted to provincial CCUs (247/929 [26.6%] vs 148/793 [18.7%], *P* < 0.001).

Descriptive baseline characteristics of patients admitted to CCUs are displayed in **Table 1**. The median age of SARI patients was 74 (58–84) years, compared with 67 (53–79) years in non-SARI patients (*P* < 0.001). Among SARI patients, 151 (38.2%) had one comorbidity and 155 (39.2%) had at least two comorbidities. The most common comorbidity among the SARI patients was chronic cardiac disease (166/395 [42.0%]), followed by chronic respiratory disease (154/395 [39.0%]) and diabetes (47/395 [11.9%]). Median time from symptom onset to hospitalization was 2 (IQR: 1–3) days in patients with SARI and 1 (IQR: 1–3) day in patients with non-SARI (*P* = 0.001).

**Table 1:** Characteristics of patients admitted to CCUs in 32 district hospitals and 16 provincial hospitals in  Viet Nam, March–July 2019

Characteristics	Patients with SARI (*n* = 395)	Patients with other diagnosis (*n* = 1 327)	*P*	Patients with SARI in district hospitals (*n* = 247)	Patients with SARI in provincial hospitals (*n* = 148)	*P*
Male gender, *n*(%)	194/395 (49.1)	780/1327 (58.8)	** < 0.001**	115/247 (45.7)	79/148 (54.9)	0.1
Age (years), median (IQR)	74 (58–84)	67 (53–79)	** < 0.001**	74 (58–85)	73 (59–83)	0.82
Days to seek care, median (range)	2 (1–3)	1 (1–3)	** < 0.001**	2 (1–3)	1 (0–3)	** < 0.001**
qSOFA score, *n*(%)						
0–1	205/395 (51.9)	886/1327 (66.8)	** < 0.001**	148/247 (59.9)	57/148 (38.5)	** < 0.001**
^3^ 2	190/395 (48.1)	441/1327 (33.2)		99/247 (40.1)	91/148 (61.5)	
Comorbidities						
Chronic respiratory disease	154/395 (39.0)	399/1327 (30.1)	** < 0.001**	109/247 (44.1)	45/148 (30.4)	** < 0.01**
Chronic cardiac disease	166/395 (42.0)	467/1327 (35.2)	**0.01**	100/247 (40.5)	66/148 (44.6)	0.64
Diabetes	47/395 (11.9)	135/1327 (10.2)	0.33	21/247 (8.5)	26/148 (17.6)	**0.01**
Chronic liver disease	11/395 (2.8)	69/1327 (5.2)	0.045	3/247 (1.2)	8/148 (5.4)	**0.02**
Chronic kidney disease	20/395 (5.1)	51/1327 (3.8)	0.28	10/247 (4.0)	10/148 (6.8)	0.34

Statistically significant *P*-values are shown in bold.

The SARI patients in district and provincial hospitals were similar in terms of the proportions of male gender (45.7% vs 54.9%, *P* = 0.1) and age (median age, 74 [IQR: 58–85] vs 73 [IQR: 59–83], respectively, *P* = 0.82) (**Table 1**). However, the duration from symptom onset to hospitalization was higher in patients with SARI presenting to district CCUs than in those presenting to provincial CCUs (median, 2 days vs 1 day, respectively).

Most district and provincial hospitals had the essential supplies and equipment to conduct diagnostic testing (e.g. chest X-ray and complete blood count) and to treat patients with SARI and sepsis. However, specific laboratory testing capacity was more available in provincial hospitals than in district hospitals, for example, for blood and sputum culture, inflammatory markers (C-reactive protein and procalcitonin), lactate, arterial blood gas and influenza A and B antigen detection (**Table 2**).

**Table 2:** Availability of supplies and intervention for management of SARI in study hospitals in Viet Nam,  March–July 2019

Supply and intervention	All hospitals (*n* = 48)	District hospitals (*n* = 32)	Provincial hospitals (*n* = 16)	*P*
Chest X-ray (%)	48/48 (100)	32/32 (100)	16/16 (100)	-
Blood culture (%)	16/48 (33.3)	3/32 (9.4)	13/16 (81.2)	** < 0.001**
Sputum culture (%)	22/48 (45.8)	9/32 (28.1)	13/16 (81.2)	**0.001**
Rapid influenza diagnostic tests (%)	21/48 (43.8)	8/32 (25.0)	13/16 (81.2)	** < 0.001**
Influenza RT–PCR test	3/48 (6.3)	0/32 (0)	3/16 (18.8)	**0.03**
Complete blood count (%)	48/48 (100)	32/32 (100)	16/16 (100)	-
C-reactive protein (%)	26/48 (54.2)	10/32 (31.2)	16/16 (100)	** < 0.001**
Procalcitonin (%)	12/48 (25.0)	1/32 (3.1)	11/16 (68.8)	** < 0.001**
Lactate (%)	18/48 (37.5)	6/32 (18.8)	12/16 (75)	** < 0.001**
Arterial blood gas (%)	19/48 (39.6)	8/32 (25)	11/16 (68.8)	** < 0.001**
Antimicrobials (%)				
Carbapenem	21/48 (43.8)	7/32 (21.9)	14/16 (87.5)	**0.04**
Cephalosporin	48/48 (100)	32/32 (100)	16/16 (100)	-
Aminoglycoside	41/48 (85.4)	26/32 (81.2)	15/16 (93.8)	0.4
Quinolone	48/48 (100)	32/32 (100)	16/16 (100)	-
Oseltamivir	12/48 (25.0)	6/32 (18.8)	6/16 (37.5)	0.29
Vasopressor (%)				
Adrenalin	48/48 (100)	32/32 (100)	16/16 (100)	-
Noradrenalin	30/48 (62.5)	14/32 (43.8)	16/16 (100)	** < 0.001**
Dopamine	41/48 (85.4)	25/32 (78.1)	16/16 (100)	0.08
Dobutamine	30/48 (62.5)	14/32 (43.8)	16/16 (100)	** < 0.001**
Corticosteroids (%)				
Hydrocortisone	27/48 (56.2)	14/32 (43.8)	13/16 (81.2)	**0.02**
Dexamethasone	27/48 (56.2)	14/32 (43.8)	13/16 (81.2)	**0.02**
Methylprednisolone	46/48 (95.8)	30/32 (93.8)	16/16 (100)	0.55
Prednisolone	31/48 (64.6)	21/32 (65.6)	10/16 (62.5)	0.83
Oxygen therapy (%)	48/48 (100)	32/32 (100)	16/16 (100)	-
Mechanical ventilation (%)	29/48 (60.4)	13/32 (40.6)	16/16 (100)	** < 0.001**
Proton pump inhibitor	44/48 (91.7)	28/32 (87.5)	16/16 (100)	0.29
Heparin	44/48 (91.7)	28/32 (87.5)	16/16 (100)	0.29

Statistically significant *P*-values are shown in bold.

**Figure 1:**
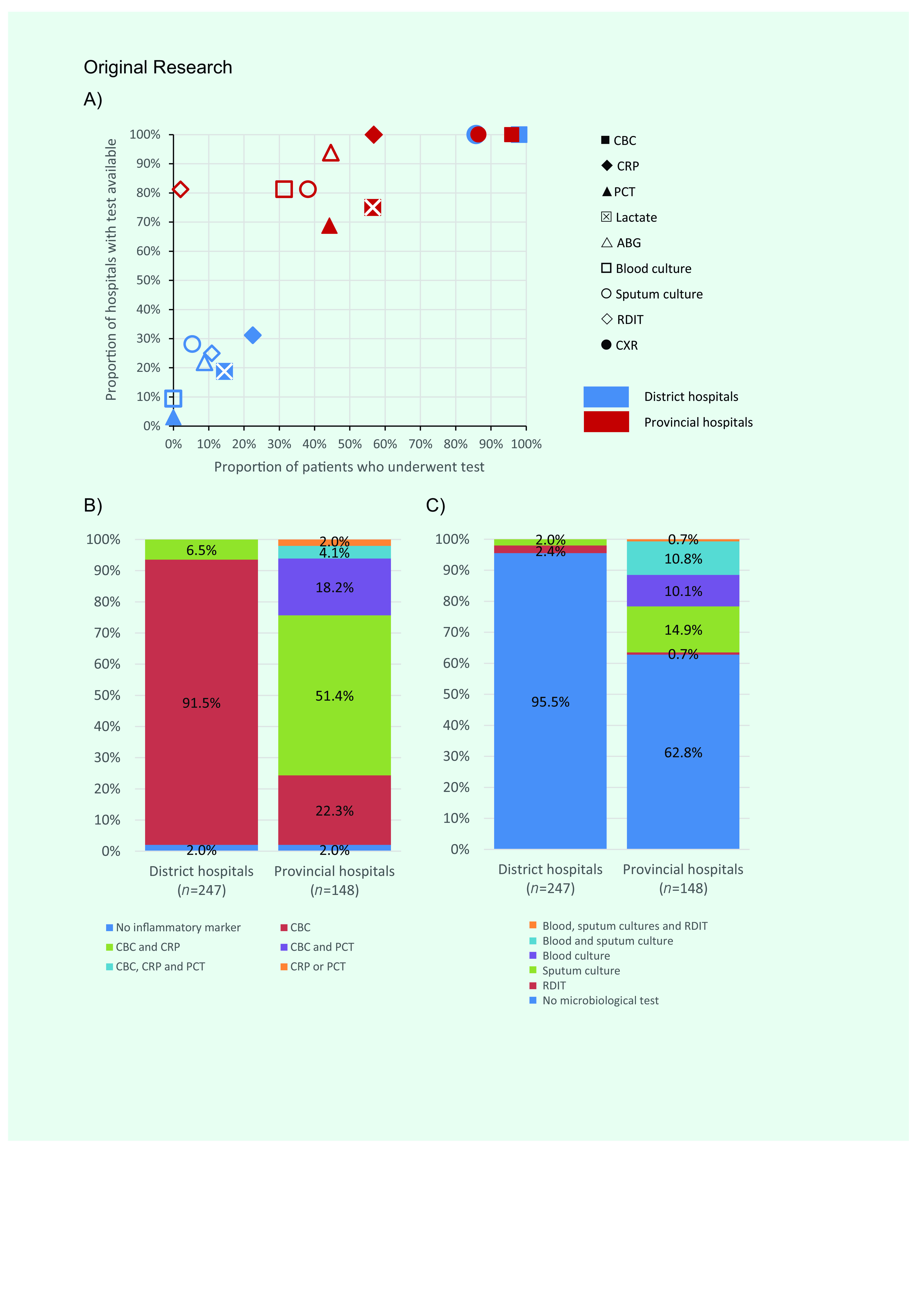
Availability and use of diagnostic tests among patients admitted to CCUs in 32 district hospitals and 16 provincial hospitals in Viet Nam, March–July 2019. A) Association between test availability and SARI patients who received each test in CCUs in district and provincial hospitals. B) Frequency of biomarker indications in patients with SARI admitted to CCUs. C) Frequency of microbiological diagnostic indications in patients with SARI admitted to CCUs

To further elucidate the impact of testing deficiency on the frequency of indicated investigations, we evaluated the association between the percentage of test availability and the proportion of SARI patients who received the corresponding test at each hospital level (**Fig. 1a**). In district hospitals, the frequency of patients who received each specific laboratory test was limited in terms of testing capacity, expressed by a significantly positive correlation (r = 0.96, *P* < 0.001). Meanwhile, in provincial hospitals, the relationship between testing capacity and frequency of testing displayed a positive trend (r = 0.36, *P* = 0.09) (**Fig. 1a**). Noticeably, among patients with SARI, 95.5% of patients in district and 62.8% of patients in provincial hospitals had no microbiological testing for etiology (**Fig. 1c**).

[**insert** Figure 1]

Among the 395 patients who met the case definition of SARI, 340 (86.1%) underwent chest X-ray, of whom 225 (66.2%) had X-ray confirmed pneumonia. However, only 8/395 patients (2%) received rapid influenza diagnostic tests, 32/395 (8.1%) received blood cultures and 44/395 (11.1%) received sputum cultures to identify the etiology of SARI. No patients were tested via polymerase chain reaction (PCR) assay for respiratory viruses, including influenza, which could be due to a deficiency of PCR machines in the participating hospitals: only three of 48 hospitals (6.3%) had the capacity to perform on-site PCR testing. In all patients with SARI diagnosis on admission, 88.4% (349/395) received empiric intravenous antibiotics within 24 hours of admission, whereas only 0.5% (2/395) received empiric oseltamivir treatment. The proportions of patients with SARI requiring oxygen therapy, invasive mechanical ventilation or vasopressors were 73.2% (289/395), 7.3% (29/395) and 4.8% (19/395), respectively, and the proportions were higher in provincial CCUs than in district CCUs (**Table 3**). The median age of patients receiving oxygen therapy and mechanical ventilation within 24 hours of admission was 76 (IQR: 63–85) and 77 (IQR: 65–88), respectively. Use of corticosteroids was common in patients with SARI (238/395 [60.3%]), particularly in district CCUs (**Table 3**). The overall rate of corticosteroid use in patients needing supplementary oxygen or invasive mechanical ventilation was 63.3% (183/289) and 65.5% (19/29), respectively, compared with 50% (51/102) in patients without respiratory support.

**Table 3:** Management of patients with SARI admitted to CCUs in 32 district hospitals and 16 provincial hospitals in Viet Nam, March–July 2019

-	All patients (*n* = 395)	District hospitals (*n* = 247)	Provincial hospitals (*n* = 148)	*P*
Antibiotics, *n*(%)	-	-	-	-
None	26/395 (6.6)	15/247 (6.9)	9/148 (6.1)	0.75
Oral route	20/395 (5.1)	11/247 (4.5)	9/148 (6.1)	
Intravenous route	349/395 (88.4)	219/247 (88.7)	130/148 (87.8)	
Oseltamivir, *n*(%)	2/395 (0.5)	2/247 (0.8)	0/148 (0)	0.53
Vasopressors, *n*(%)	19/395 (4.8)	2/247 (0.8)	17/148 (11.5)	** < 0.001**
Corticosteroids, *n*(%)	238/395 (60.3)	168/247 (68.0)	70/148 (47.3)	** < 0.001**
Oxygen therapy, *n*(%)	289/395 (73.2)	160/247 (64.8)	129/148 (87.2)	** < 0.001**
Mechanical ventilation, *n*(%)	29/395 (7.3)	7/247 (2.8)	22/148 (14.9)	** < 0.001**
Heparin, *n*(%)	27/395 (6.8)	7/247 (2.8)	20/148 (13.5)	** < 0.001**
Proton pump inhibitors, *n*(%)	174/395 (44.1)	92/247 (37.2)	82/148 (55.4)	** < 0.001**

The overall 7-day mortality in patients presenting to CCUs was 6.6% (26/395) (**Appendix Fig. 1**). The 7-day mortalities in patients initially admitted to district and provincial CCUs were 10/247 (4%) and 16/148 (10.8%), respectively (*P* < 0.001). The 7-day mortality of all SARI cases was similar to the mortality of those with other diagnoses (26/395 [6.6%] vs 79/1327 [6.0%], respectively, *P* = 0.65). The median age of patients who died was 74 (IQR: 60–84) for SARI cases and 72 (IQR: 59–84) for patients with non-SARI diagnoses.

The median time to death for SARI cases was 3 days (IQR: 2–5). Multivariate Cox proportional hazard regression analysis indicated that septic shock (hazard ratio [HR]: 3.5, 95% confidence interval [CI]: 1.23–9.96) and qSOFA score (*3*)2 (HR: 3.41, 95% CI: 1.25–9.34) within the first 24 hours of CCU admission were associated with death (**Table 4**).

**Table 4:** Cox proportional hazards model of factors associated with 7-day mortality among SARI patients admitted to CCUs in 32 district hospitals and 16 provincial hospitals in Viet Nam, March–July 2019

Variable	Hazard ratio (95% CI)	*P*
Age (1-year increment)	1 (0.97–1.02)	0.78
Male gender	0.59 (0.26–1.31)	0.19
Initial admission at secondary hospitals	1.59 (0.67–3.75)	0.29
Comorbidities	6.21 (0.78–49.44)	0.08
Septic shock within first 24 hours of admission	3.5 (1.23–9.96)	**0.02**
Oxygen or mechanical ventilation within first 24 hours of admission	1.17 (0.31–4.48)	0.82
qSOFA on admission (*3*) 2	3.41 (1.25–9.34)	**0.02**
X-ray confirmed pneumonia	0.69 (0.29–1.62)	0.39

Statistically significant *P*-values are shown in bold.

## Discussion

Our study shows that SARI remains a burden on the Vietnamese health care system. A considerable proportion of SARI cases (22.9%) were admitted to CCUs, and 7-day mortality (6.6%) was not negligible in the pre-COVID-19 era. Laboratory testing for SARI was severely limited in the district hospitals and underused in the provincial hospitals included in this study.

Previous studies in developing countries demonstrated that SARI was common among patients admitted to emergency departments (range of about 20–30%). (*25*, *26*) In a surveillance study of 15 sites in Viet Nam during 2006–2010, the hospital admission rates in outpatients presenting with influenza-like illness (ILI) – defined as a measured temperature of 38 °C or more and cough and/or sore throat – was 9.3%. Of 6516 outpatients with ILI tested for influenza by PCR, 22% were positive. (*27*) In a study of hospital admissions in a tertiary paediatric hospital in Hanoi during 2007–2014, pneumonia and bronchitis were the leading causes and accounted for 24.5% and 19.1% of all emergency visits, respectively. (*28*) In 2016, SARI surveillance on 4003 specimens revealed that 20.2% were positive for influenza virus and 41.8% were positive for at least one non-influenza respiratory virus (including 16.2% respiratory syncytial virus, 13.4% rhinovirus, and 9.6% adenovirus and other viruses). (*15*) During the study period, the participating hospitals were not actively involved in SARI sentinel surveillance, and no data were reported.

One study conducted at a provincial hospital in Viet Nam in 2009–2010 demonstrated a case mortality rate of 9.8% among hospitalized patients with community-acquired pneumonia. (*17*) In our study, the number of SARI cases admitted to CCUs was higher, but the mortality rate was lower at 6.6%. This may be due to the greater number of patients in district hospitals, where the clinical severity of cases tends to be milder, and to early mortality being assessed at day 7 after admission, which can lead to underestimation of the mortality rate in CCUs and hospitals.

In our study, SARI cases tended to be older and had more chronic cardiovascular and respiratory comorbidities than the general population. This is concordant with previous studies in which SARI cases tended to be associated with risk factors including older age and underlying heart and pulmonary diseases. (*29*, *30*)

We found an apparent disparity in laboratory testing capacity between district and provincial hospitals in Viet Nam. Although the diagnosis of respiratory infections is more commonly based on physical examination, chest imaging and identification of pathogens are key to clinical management, especially in critically ill patients. Laboratory testing also contributes to identifying and preventing issues with antimicrobial resistance. (*31*, *32*) A SARI surveillance study in Egypt demonstrated that patients for whom pathogens were identified had a significantly lower rate of intensive care unit admission, length of hospital stay and overall mortality than those with unknown etiology. (*33*) Although the predominant pathogens in SARI cases are presumably viruses, especially influenza (up to 50% of tested respiratory samples from previous surveillance in Viet Nam), (*15*, *27*, *34*) strengthening laboratory capacity to identify causal pathogens is critically important for the management of not only SARI but also of other emerging and re-emerging diseases, considering the current burden of SARI cases in CCUs in Viet Nam. In regards to laboratory testing, in addition to microbiological identification tests (blood culture, sputum culture or viral PCR for respiratory tract specimens), other investigations recommended for severity assessment, antibiotic de-escalation and mortality prediction in SARI include blood gas analysis or inflammatory and sepsis markers (C-reactive protein, procalcitonin and lactate). (*35*-*39*) The shortage and underuse of these tests in our study reinforces the need to develop a care bundle for SARI management to further improve the quality of care in LMICs.

We found that 93.4% of patients in our study were given empiric antibiotics within the first 24 hours of admission, but only a small number of patients received antiviral drugs. For patients with SARI presenting to CCUs, the use of empiric antimicrobials on admission is reasonable and recommended. (*31*) Corticosteroids were more commonly used in district hospitals than in provincial hospitals, although international guidelines advise against routinely using corticosteroid therapy in patients with community-acquired pneumonia. (*31*)

In our 2017 survey, we noted a shortage of supplies and equipment in the district hospitals compared with provincial hospitals and a lack of ventilators at both hospital levels. (*18*) In this study, we reaffirmed that – in addition to the availability of equipment – supply of and access to laboratory tests for critical care in district hospitals were still insufficient for SARI management. The current SARS-CoV-2 pandemic has highlighted vulnerabilities of the critical care system for SARI management caused by a shortage of supplies, especially ventilators, even in developed countries. (*40*) Under the current situation of COVID-19, accurate diagnosis of SARS-CoV-2 is solely based on nucleic acid amplification tests, which have major capacity constraints in almost all CCUs in district hospitals. Hence, both the limitations of laboratory and supply capacities are major obstacles for CCUs in district hospitals in Viet Nam to cope with COVID-19.

There were several limitations in our study. First, because the study hospitals were selected by convenience sampling, the findings were not representative of the capacity of the health care system nationwide, although we believe it reflected the general situation in Viet Nam. Second, for the purposes of this study, SARI was defined using clinical symptoms. Without a doctor’s justification, there may have been bias in actual diagnosis and indications for investigations (i.e. the hospital doctors may have another differential diagnosis that indicated different practices for laboratory test orders).

In conclusion, our study reported a high rate of CCU admission among SARI patients in selected district and provincial hospitals in Viet Nam. With the current insufficiencies in diagnostic and treatment capacity in district hospitals and underuse in provincial hospitals, it is recommended that a standardized protocol for SARI management in resource-constrained settings be developed to improve quality of care.
