# Supplementary material for: Capacity and use of diagnostics and treatment for patients with severe acute respiratory infections in the pre-COVID-19 era in district and provincial hospitals in Viet Nam
Source: Western Pac Surveill Response J. 2021 Nov 30;12(4):1–9. doi: 10.5365/wpsar.2021.12.4.835 (PMC8873919; doi:10.5365/wpsar.2021.12.4.835)
Supplement: Supplementary file 1 [file wpsar-12-835-s001.pdf]

Appendix Fig. 1. **Flow chart of study population**

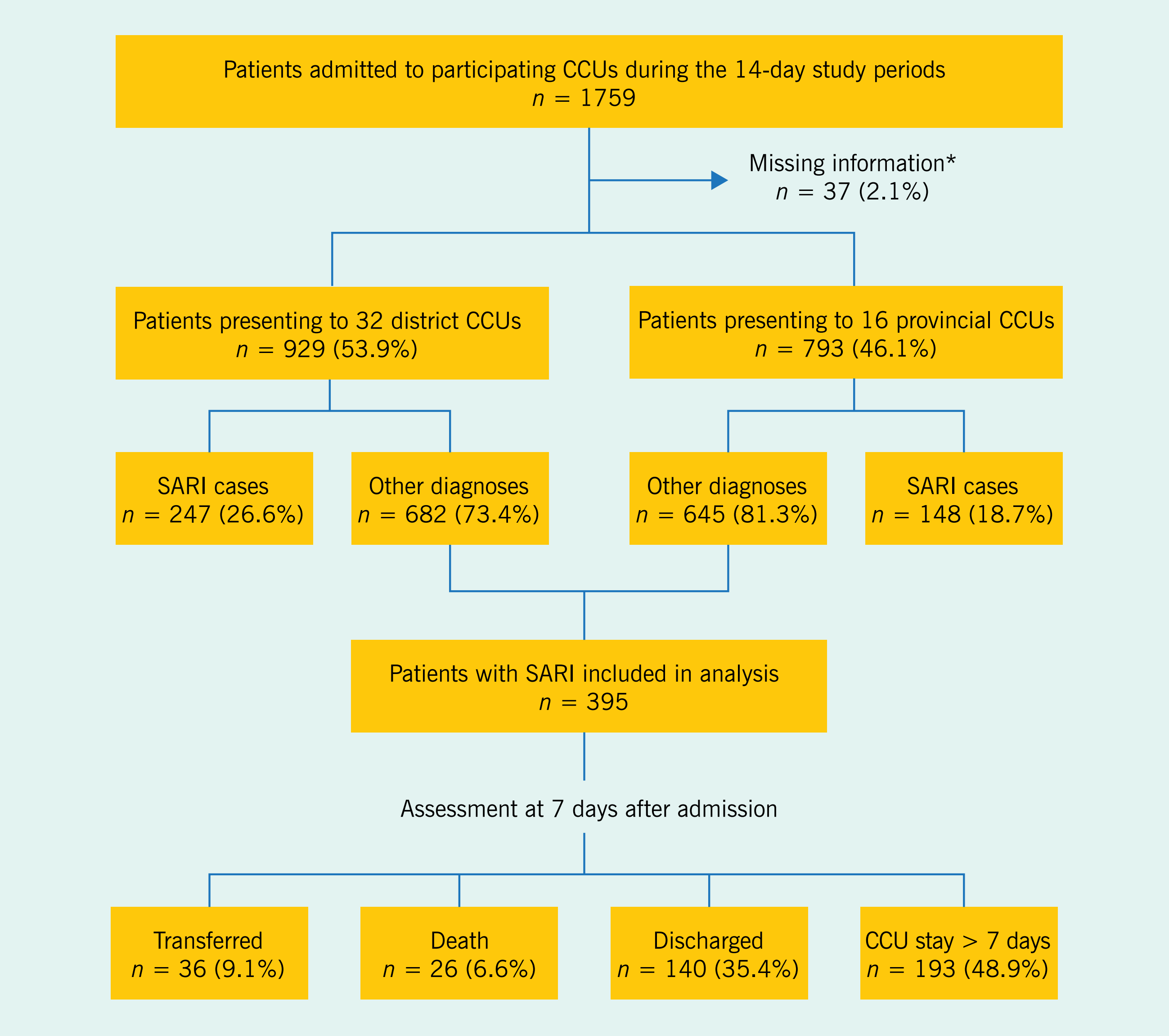

* Missing information on diagnosis and/or time of symptom onset.
